# Understanding delays in breast cancer diagnosis in Africa: Key insights and contributing factors

**DOI:** 10.1002/ijc.70008

**Published:** 2025-06-20

**Authors:** Liza A. Hoveling, Lynn P. Heuken, Thachita Harfst, Melinda S. Schuurman, Kristel M. van Asselt, Sabine Siesling, Christina Bode

**Affiliations:** ^1^ Department of Health Technology and Services Research Technical Medical Centre, University of Twente Enschede The Netherlands; ^2^ Department of Psychology, Health and Technology University of Twente Enschede The Netherlands; ^3^ Department of Research and Development Netherlands Comprehensive Cancer Organisation (IKNL) Utrecht The Netherlands; ^4^ Department of General Practice and Nursing Science UMC Utrecht Utrecht The Netherlands

**Keywords:** African countries, breast cancer, Bronfenbrenner's ecological model, diagnostic delays, low and middle income countries

## Abstract

Africa has the highest age‐standardized breast cancer (BC) mortality rates, largely due to diagnostic delays. Therefore, this scoping review aims to identify individual‐level factors that contribute to diagnostic delay of BC in African women. We conducted a global scoping review on cancer diagnostic delays in women, following PRISMA‐ScR guidelines. In this scoping review, diagnostic delay is defined as the time from first symptom recognition to pathological diagnosis. Qualitative and quantitative studies involving cancer patients or healthcare professionals published between 2018 and November 28, 2023, were included. We searched PubMed/MEDLINE and Scopus, excluding non‐English studies and those focused solely on screening. Two reviewers independently screened titles, full texts, and extracted data. Disagreements were resolved by discussion. Consultations followed Arksey and O'Malley's framework, with input from a general practitioner, psychologist, and epidemiologist. Factors were classified using Bronfenbrenner's ecological model to analyze BC diagnostic delays in Africa. Of 9699 studies, 128 were relevant; 30 focused on African BC patients. Delays were linked to microsystem factors: lack of awareness, fear, young age, low education, finances, mesosystem factors: family duties, limited access, delayed care, symptom disclosure, exosystem factors: traditional healers, mistrust, referral inefficiencies, and macrosystem factors: religious beliefs, education gaps, cultural norms. Diagnostic delays in women with BC in Africa are mainly due to low awareness, cultural beliefs, and reliance on traditional healers. Expanding current interventions and integrating them into healthcare systems, along with engaging religious leaders, is important. Future research should focus on culturally tailored strategies to improve early detection and outcomes.

## Graphical abstract

What's new?

Despite lower incidence rates, Africa has the highest age‐standardized breast cancer mortality rates globally. Using Bronfenbrenner's ecological model to classify multilevel influences on the individual, this scoping review shows that a lack of knowledge about breast cancer is the primary factor contributing to breast cancer diagnostic delays in African women. Cultural beliefs attributing breast cancer to spiritual causes, financial barriers, and reliance on healthcare providers other than academically trained doctors further increase these delays. The findings provide actionable insights for culturally tailored health policy measures and interventions, supporting integration of awareness campaigns into existing healthcare systems.

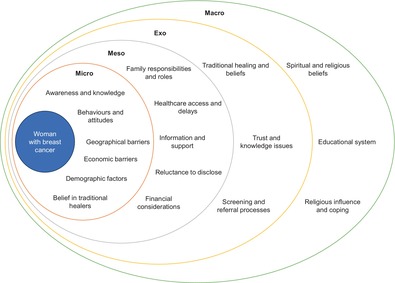

**List of Abbreviations**
BC: breast cancer
GDP: gross domestic product
GP: general practitioner
LMICs: low‐ and middle‐income countries
OSF: Open Science Framework
PRISMA‐ScR: Preferred Reporting Items for Systematic Reviews and Meta‐Analyses for Scoping Reviews

## 1 INTRODUCTION

Breast cancer (BC), the most diagnosed cancer globally, accounted for 12% of all cases in 2022.^1^ In Africa, despite lower incidence, BC mortality is highest due to late‐stage presentation and incomplete treatment.^1^, ^2^ Diagnostic delay often leads to advanced‐stage BC and poorer outcomes.^3^, ^4^ Although existing reviews^5^ touch on delay, few provide a recent, Africa‐specific analysis. Our review fills this gap by analyzing socio‐cultural, behavioral, and systemic factors using Bronfenbrenner's ecological model.^6^ Definitions of diagnostic delay vary, typically covering the period from symptom recognition to pathological diagnosis.^5^ Typically, diagnostic delay is described as the time from initial symptom recognition to pathological diagnosis, as also stated in Weller et al.'s Aarhus statement.^5^ While definitions of diagnostic delay may differ slightly (e.g., start point at first clinical consultation or referral), this review uses the common definition of diagnostic delay, starting with symptom recognition by the patient and ending with the pathological diagnosis^7^ The WHO recommends a diagnostic interval under 60 days, a target many African countries struggle to meet.^7^, ^8^

Factors of diagnostic delay can be examined using an ecological model, which shows various layers of influence on behavior. This model could help to explore how factors at different levels (micro‐, meso‐, exo‐, and macrosystem level) influencing the individual (e.g., individual‐level factors) contribute to delays in cancer diagnosis. Research on diagnostic delays in cancer within low‐ and middle‐income countries (LMICs), particularly in Africa, has already identified several factors contributing to late diagnoses. These factors can be mapped across different levels of the ecological model. At the microsystem level, individual behavioral factors such as health‐seeking behaviors^9^ and belief in traditional medicine^10^ have been studied. Emotional and cognitive aspects, such as health literacy, awareness,^11^ and beliefs about cancer, also play a role in when individuals decide to seek medical attention.^12^ At the mesosystem level, social influences like family support, community norms, and competing responsibilities (e.g., work, childcare) have been identified as factors affecting healthcare access.^13^, ^14^ The exosystem level involves broader contexts such as education, employment status, availability of healthcare facilities, and regional inequalities in healthcare infrastructure which impact access to healthcare services.^14^ At the macrosystem level, policy factors, insurance coverage, government investments in healthcare (e.g., screening programs and self‐detection education), societal norms, cultural values, and economic conditions shape overall attitudes toward health and illness, influencing delays in seeking and receiving a cancer diagnosis.^15^

This review categorizes individual‐level factors for diagnostic delay in African women with BC using Bronfenbrenner's ecological model^6^ to inform targeted interventions.

## 2 METHODS

This scoping review expands on Hoveling et al.^16^ and follows Preferred Reporting Items for Systematic Reviews and Meta‐Analyses for Scoping Reviews (PRISMA‐ScR) guidelines.^17^, ^18^

### 2.1 Literature search strategy

Separate searches in PubMed/MEDLINE and Scopus using the queries listed in Tables 1, S1, and S2 were performed. The protocol was registered on the Open Science Framework (OSF) (https://osf.io/pwgt8). Strategy included relevant cancer‐related terms (e.g., oncology, carcinoma) and delay‐related terms (e.g., postponed, disrupted). The search process was refined with information specialists, and pilot tests ensured the inclusion of all previously identified studies. Search terms did not have any geographical restrictions. The search strategy, therefore, was not limited geographically, but the focus was applied later in the sub‐analysis based on data derived from the previous scoping review.^16^ The final searches were completed on November 28, 2023.

**TABLE 1:** Search queries (also registered on the Open Science Framework (OSF) (https://osf.io/pwgt8) used for titles, abstracts, and keywords in the database search performed on 28th November, 2023.

Database	Query
PubMed/MEDLINE	((“diagnos*”[Title/Abstract] AND “delay*”[Title/Abstract]) OR (“care”[Title/Abstract] AND “delay*”[Title/Abstract]) OR “patient delay”[Title/Abstract] OR “presentation delay*”[Title/Abstract] OR “timely diagnos*”[Title/Abstract] OR “primary care delay*”[Title/Abstract] OR “late diagnos*”[Title/Abstract]) AND (“cancer”[Title/Abstract] OR “tumor”[Title/Abstract] OR “neoplasm”[Title/Abstract] OR “malignan*”[Title/Abstract] OR “carcino*”[Title/Abstract] OR “oncolog*”[Title/Abstract] OR “sarcoma”[Title/Abstract] OR “leukemia”[Title/Abstract] OR “lymphoma”[Title/Abstract] OR “melanoma”[Title/Abstract] OR “blastoma”[Title/Abstract]) AND (“determinant*”[Title/Abstract] OR “influence*”[Title/Abstract] OR “barrier*”[Title/Abstract] OR “factor*”[Title/Abstract] OR “reason*”[Title/Abstract]) AND (“women”[Title/Abstract] OR “woman”[Title/Abstract] OR “female”[Title/Abstract] OR “gender”[Title/Abstract] OR “sex”[Title/Abstract] OR “breast”[Title/Abstract] OR “cervi*”[Title/Abstract] OR “uter*”[Title/Abstract] OR “endometr*”[Title/Abstract] OR “ovar*”[Title/Abstract] OR “vulv*”[Title/Abstract])
Scopus	(TITLE‐ABS‐KEY((diagnosis AND delay) OR (care AND delay) OR “patient delay” OR “presentation delay” OR “timely diagnosis” OR “primary care delay” OR “late diagnosis”)) AND (TITLE‐ABS‐KEY[cancer OR tumor OR neoplasm OR malignant OR carcinoma OR oncology OR sarcoma OR leukemia OR lymphoma OR melanoma OR blastoma]) AND (TITLE‐ABS‐KEY [determinant OR influence OR barrier OR factor OR reason]) AND (TITLE‐ABS‐KEY [women OR woman OR female OR gender OR sex OR breast OR cervix OR uterus OR endometrium OR ovary OR vulva])

### 2.2 Inclusion and exclusion criteria

Eligible for analysis were both qualitative and quantitative studies involving oncological patients, women or healthcare professionals, focusing on patient‐related factors, behaviors, cognitive aspects, emotional responses, and diagnostic processes. The inclusion of both qualitative and quantitative studies provides a comprehensive understanding of diagnostic delays, where quantitative studies provide measurable prevalence data and statistical associations, while qualitative studies offer deeper insights into patient experiences and social determinants influencing healthcare‐seeking behavior. Focus was on studies from LMIC African countries, because of the assumption that healthcare systems in these settings would be homogeneous in terms of resources and access to care. Studies that involved African women outside of Africa were excluded, while non‐Black women within Africa, were not excluded. Exclusions applied to studies not meeting these criteria, not in English or Dutch, or solely focused on screening. Non‐original research (e.g., reviews, commentaries, editorials) was also excluded. The review focused on studies published between 2018 and 2023 to ensure relevance.

### 2.3 Study screening

Titles of all identified studies were screened by L.A.H. after duplicate removal, followed by full‐text screening. M.S. independently reviewed titles and full texts for 20% of records from 2018 to 2023. Reviewers agreed on 86% of studies, with the remaining 14% resolved through discussion. Six studies (13%) required additional review by C.B., K.M.A., and S.S.

### 2.4 Data extraction

Data extraction was performed by L.A.H., with M.S. independently extracting data from 20% of the studies. Consensus was reached before analysis. Extracted details included: first author, publication year, country, gross domestic product (GDP) per capita (from an external resource^19^), cancer detection method (e.g., self‐screening, interpreted according to each study's definition), study type, study objectives, population characteristics, study center, year of inclusion, study design, type of delay assessed included patient, primary care, secondary care, diagnostic (unspecified), and presentation delays. “Patient delay” is the time from symptom recognition to first healthcare contact; “primary care delay” from first contact to referral; and “secondary care delay” from referral to diagnosis. “Presentation delay” often overlaps with patient delay but may vary by study. In this review, we define “diagnostic delay” as the time from symptom recognition to pathological diagnosis, aligning with the full diagnostic interval per Weller et al.'s Aarhus statement.^5^ While the WHO distinguishes between patient and diagnostic delays, we adopt the broader definition covering the entire period from symptom onset to diagnosis. Included studies varied in defining start and end points; we noted which part of the diagnostic pathway each study addressed.

### 2.5 Consultation

Following Arksey and O'Malley,^18^ we consulted a GP, psychologist, and epidemiologist for feedback on preliminary findings, which were incorporated into the results.

### 2.6 Data analysis

Sufficient African BC studies allowed classification of delay factors using Bronfenbrenner's ecological model,^6^ facilitating an overview on multiple levels.

## 3 SEARCH RESULTS

**FIGURE 1:**
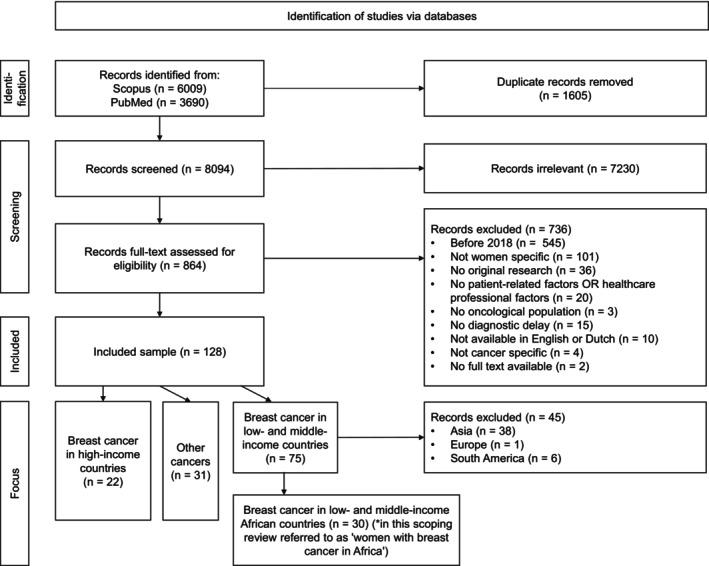
PRISMA flowchart depicting the process of study selection and the rationale for exclusions during full‐text screening.

The database search by Hoveling et al.^16^ identified 9699 records, with 1605 duplicates removed. After title screening, 7230 of the 8094 unique records were excluded based on exclusion criteria. A total of 128 studies underwent full‐text examination. After reviewing 128 studies, articles examining African dynamics of diagnostic delay in BC were categorized by L.P.H. and T.H., leading to the selection of 30 studies. Figure 1 presents a flow diagram of the screening and selection process.

## 4 RESULTS

### 4.1 General overview of the included studies

**FIGURE 2:**
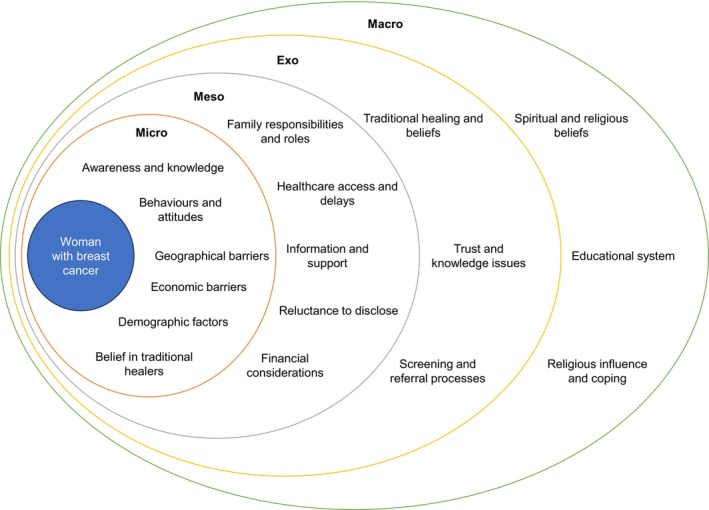
Ecological system of women with breast cancer and diagnostic delay in Africa.

Thirty studies focusing on individual‐level factors in diagnostic delay were included (Tables S3 and S4). Primarily focused on symptomatic BC detection. Seventeen were quantitative,^20^, ^21^, ^22^, ^23^, ^24^, ^25^, ^26^, ^27^, ^28^, ^29^, ^30^, ^31^, ^32^, ^33^, ^34^, ^35^, ^36^ 11 qualitative,^37^, ^38^, ^39^, ^40^, ^41^, ^42^, ^43^, ^44^, ^45^, ^46^, ^47^ and two mixed‐methods.^48^, ^49^ Applying the Bronfenbrenner ecological model^6^ to the findings, various individual‐level factors interact within their environment, alongside external influences and broader cultural and societal norms were categorized (Figure 2).

### 4.2 Microsystem

Most important and frequently mentioned intrapersonal factor causing diagnostic delays in BC is the lack of awareness and knowledge, particularly among women with BC in Africa regarding the disease and its symptoms.^25^, ^47^, ^48^ An Ethiopian study showed that, before their diagnosis, patients had no knowledge on the disease.^46^ Gebremariam et al.^20^ found that in their sample every third of the 441 Ethiopian patients has never heard of BC before being diagnosed. Additionally, they argued that the women did not seek medical help because their lack of knowledge caused a misinterpretation of the severity of their condition. Moreover, they showed that the benefits that originate from early treatment were unknown to the patients, too.

Primary, demographic factors such as age and education level are influential. Magara et al.^49^ found that women under 30 years old had the least knowledge of BC, while Rayne et al.^34^ found similar results with an age lower than 45 years. Interestingly, also many other studies argued for the correlation between young age and lack of awareness.^24^, ^26^, ^27^, ^30^, ^35^ Demographic exploration of the population showed that women with less education had more diagnostic delay.^23^, ^34^, ^49^ Additionally, most patients in these studies from Egypt and Ethiopia who experience delay are illiterate.^24^, ^29^ Ismail et al.^24^ showed that, when comparing university and high school graduates, university graduates had less patient delay.

Lack of awareness about BC and its symptoms contributes to delayed diagnosis, often due to misattribution of symptoms.^41^ For instance, the misconception that BC typically causes pain leads to incorrect self‐diagnosis, as a lack of pain is more common.^39^, ^42^ Symptoms like lumps or swelling are frequently misinterpreted as being caused by sunstroke, aging, oral contraceptives, a history of breast diseases, or breastfeeding.^21^, ^41^ Additionally, infrequent self‐screening, linked to poor knowledge about BC and its management,^24^ further delays diagnosis. Despite awareness, women rarely practiced mammography or self‐exams due to missing information about early detection benefits.^23^ Even when abnormalities were discovered, as Gebremariam et al.^20^ and Tesfaw et al.^46^ reported, it was often accidental, such as while undressing or showering, rather than through intentional or population screening.

Besides lack of self‐screening, fear and stigma surrounding diagnosis and treatment also contribute to delay.^32^ A study by Tesfaw et al.^46^ suggested that patients' fear of medical procedures contributed to their delay in seeking early medical care for breast issues. This fear is often grounded in the communities' misconceptions such as death from surgery or infertility after mastectomy. Other types of fear related to cancer include a fatalistic perspective, anxiety about receiving a cancer diagnosis, and concerns about public stigma. Patients often worry about being labelled within their communities, as well as the potential for gossip and negative comments. In particular, Tanzania's history of poor cancer outcomes has contributed to a widespread fear, directly linking cancer with death.^43^

Strong belief in traditional healers also contributes to delay. Traditional medicine is often preferred due to its accessibility, affordability, and perceived quick relief, despite lacking evidence‐based treatment.^41^, ^43^ Many believe prayers and rituals provide a cure, delaying medical care until traditional practices fail. Distrust in health professionals, coupled with long‐standing cultural reliance on traditional healers, further hinders timely diagnosis.

Costs of treatment, transportation, and healthcare are the most mentioned aspects of the African healthcare system and receive explaining value for diagnostic delay when considering the financial insecurity of the African population.^33^, ^47^ Many patients emphasize how burdensome the long travels to the few available cancer centers are and that they are often accompanied by high costs, which they cannot afford.^34^, ^44^, ^49^ Patients and their families who struggle to gather funds for recommended tests, travel, or temporary accommodation close to a cancer center lead to even further delay of a diagnosis.^43^ In the Tanzanian communities, for instance, the majority do not have health insurance, causing them to return to their hometown to earn more money before being able to continue their treatment.

Another study by Tesfaw et al.^29^ suggested that women from rural areas were four times more likely to experience delays in seeking medical attention compared to those residing in urban areas. In the province of Mashonaland South in Zimbabwe, over 25% of the population resided more than 10 km from the nearest clinic, and up to 75% were unable to afford necessary medications or travel expenses.^49^ Additionally, most patients could not afford effective private health care and generally relied on public healthcare. The financial insecurity of patients clearly hindered the timeliness of cancer diagnoses due to the costs of qualitative healthcare, treatment, and transportation.^33^, ^41^, ^42^, ^43^, ^48^

### 4.3 Mesosystem

A factor for and against diagnostic delay in women in Africa is responsibilities to their family. Being a housewife comes with responsibilities that many women are afraid not to be able to fulfil anymore if they receive the diagnosis and treatment.^41^, ^44^, ^45^ Soliman et al.^44^ reported that women feared that BC can affect their performance as mothers.^21^ Due to priorities and responsibilities children and a household bring along, women postpone visits to health care centers and even pay less attention to bodily signs of BC.^39^, ^41^ The women fear that this diagnosis interferes with their daily functioning and, therefore, see the diagnosis as a risk and threat.^37^ Swinny et al.^45^ expanded this factor in terms of disclosure. Not only is delay caused by the wish to care for loved ones, but also the missing motivation to disclose symptoms to them. Moreover, Zahfir et al.^31^ observed that married women experienced more diagnostic delay. Their argumentation centered around fear of divorce and missing to fulfil responsibilities as a consequence of a BC diagnosis. Lastly, the family as well as the church and peers, can potentially provide information, however, they often do not have correct or complete information about BC, either.^39^, ^47^, ^49^ Tesfaw et al.^46^ showed that the patients' families lacked knowledge of the potential risks of BC.

Having these risk factors in mind, having a family could also reduce diagnostic delay. Research shows that the family has major functions as a support system and can therefore promote help‐seeking behavior in women with BC symptoms.^41^, ^43^ Husbands particularly play an important role in the decision‐making on healthcare visits. Women often passively participate in the decision‐making regarding healthcare visits since they cannot financially afford any treatment and rely on the financial support of their husband.^38^ While friends and family can help patients, their help relies on information on symptom complaints provided by the women. However, considering that women's disclosure is affected by their responsibility and functioning within a family, persuasion and support are limited when the women do not express symptoms.^45^

### 4.4 Exosystem

Due to limited access to formal healthcare services in certain regions, many patients within African communities initially seek help from traditional healers. In Tanzania, traditional services are preferred for their accessibility and perceived quick relief.^43^ However, they are not grounded in evidence‐based treatment and generally involve prayers as a method of healing. Not only do the prayers and religions offer comfort, but many individuals believe that these are a cure for their illness (aligned with the microsystem). Some studies in other sub‐Saharan countries found that the use of alternative therapies was a common factor contributing to the delayed diagnosis of BC.^43^ Similarly, Gebremariam et al.^41^ found that the prevalent use of traditional medicine was the primary cause of late diagnosis among participants from Ethiopia. They further stated that some BC patients only sought medical care after the traditional practices failed. Preferences for traditional medicine are often caused by a lack of trust in health professionals (aligned with the microsystem). Long‐standing family and community reliance on traditional healers and witchcraft exacerbates these suspicions of conventional medicine, delaying referrals for conventional investigation until it is too late for effective treatment.

Patients' preference for traditional healers often stems from a lack of trust in the healthcare system, partly due to perceived or actual gaps in the knowledge and training of health professionals.^47^ Despite BC awareness, misconceptions and poor self‐screening contribute to delay.^23^ Even among health professionals, self‐screening practices remain inconsistent. For example, Halmata et al.^23^ and Simo et al.^28^ found that in Cameroon, only 24.7% of professionals practiced monthly breast self‐examination (BSE), indicating that knowledge alone does not always translate into regular screening behavior.

As a result of the insufficient knowledge and education among some health professionals, many patients experience prolonged referral processes and extended waiting times before receiving a final diagnosis. In fact, in a qualitative study by Gakunga et al.,^40^ participants reported receiving misinformation and false recommendations, leading to delays in obtaining a final diagnosis, even for those with a family history of BC. Lack of training among professionals often leads to misinformation, reinforcing patient unawareness.^23^ Moreover, receiving a diagnosis from a health professional often occurs in an insensitive manner and without the opportunity for counselling, which may stem from a heavy workload and lack of knowledge of the healthcare professional.

### 4.5 Macrosystem

At the broadest cultural level, diagnostic delay can also be attributed to the religious conceptualization of BC.^34^ Many studies showed that the causal inference women draw upon BC was explained by spiritual penalty and sin.^38^, ^39^, ^46^ Some women believed divine punishment made death inevitable regardless of diagnosis.^34^ Moreover, they favored dying with a breast over living without one and being “less of a woman.” These religious inferences about BC reduce the perceived need for diagnosis and treatment in BC patients. Furthermore, their spiritual argumentation of the etiology of BC causes health‐seeking behavior^38^, ^46^ is the traditional spiritual circuit. These women believe that faith in God and the use of holy water will heal BC, with medical treatment only being considered as a last resort, often when it is already too late.^34^, ^46^ Since Africa has many different religions, the influences of these conceptualizations differ. Martei et al.^42^ reported that these beliefs are stronger for Muslims than Christians since they found that Christians seek sanctuary in the Church to cope with the societal stigma of BC.

## 5 DISCUSSION

Our study highlights that a lack of awareness and knowledge about BC and its symptoms is the primary factor contributing to diagnostic delays in African countries. Cultural beliefs attributing BC to spiritual causes, financial barriers, and reliance on healthcare providers other than academically trained doctors (e.g., traditional healers, faith healers or herbalists) further increase these delays.

### 5.1 Microsystem

Diagnostic delays in BC in Africa are primarily due to low awareness and misinterpreted symptoms. Other contributing factors include young age, low education, fear, stigma, and financial barriers, particularly in rural areas. We propose intervention strategies. These include developing targeted educational programs to increase awareness of BC, focusing on symptom recognition and early detection benefits. Local community leaders and healthcare workers can help disseminate accurate information and dispel misconceptions. Improving access to healthcare, providing financial support for travel and treatments, and offering psychosocial support are important. Training women in effective self‐examination practices can also enhance their awareness and diagnostic skills. Self‐examination remains debated as a screening method. Several initiatives, one in Ethiopia with 525 individuals, and one in Tanzania with 1129 individuals, are already in progress in Africa and other LMICs. In some areas, community‐based awareness campaigns are educating women about BC symptoms and the importance of early detection.^50^, ^51^ Mobile screening units are being deployed to reach women in remote areas, making screening services with mammography more accessible.^51^ Initiatives such as “Know Your Lemons” are also being launched, focusing on BC self‐screening in countries like Nigeria.^52^ Additionally, programs aimed at training healthcare workers are being implemented to improve the quality of care and provide accurate information to women at risk.^50^

### 5.2 Mesosystem

Our review shows that family influence encourage BC diagnosis in women in Africa. Responsibilities at home and fear of divorce often lead women to delay seeking help, family and community support can promote timely care. However, misinformation within families and communities frequently hinders early diagnosis. Raising BC awareness among women and their families, particularly by providing accurate information to husbands and other key family members, is needed. Empowering women financially supports autonomous health decisions. In parts of Africa and other LMICs, initiatives like community‐based awareness campaigns and mobile screening units are in progress.^50^, ^51^, ^53^ Programs train healthcare workers to better support women.^54^, ^55^

### 5.3 Exosystem

We showed that, in African communities, patients often turn to healthcare providers other than academically trained doctors due to their accessibility, affordability, and quick relief. Distrust of healthcare professionals, coupled with inconsistent self‐screening and a lack of knowledge among healthcare workers, further contributes to delayed diagnosis and treatment. Such providers may be a necessary alternative when formal care is inaccessible. Structural issues, such as shortages of biopsy professionals and diagnostic equipment, may leave patients with limited options, making traditional health a replacement rather than a deliberate choice over conventional care. Systematic healthcare issues shape individual choices. Promoting collaborations between healthcare providers other than academically trained doctors and healthcare workers to encourage early referrals to conventional care is important. Healthcare worker training in BC awareness and communication is needed to reduce misinformation. Awareness campaigns should address delays and misconceptions. The introduction of psychosocial support and counselling for patients (and their families) who receive a diagnosis can improve the quality of care and help patients make timely treatment decisions.^56^, ^57^, ^58^, ^59^, ^60^ In Africa, studies show that collaboration between healthcare providers other than academically trained doctors and orthodox practitioners to provide coordinated care and ensure that patients receive the best possible care is important.^61^, ^62^, ^63^ Additionally, programs are being developed to better train healthcare workers in BC care,^54^, ^55^ and public awareness campaigns are being launched to improve awareness and screening.^64^

### 5.4 Macrosystem

Our study found that religious beliefs largely contribute to BC diagnostic delays in Africa. Spiritual interpretations of BC delay medical care. The perception of living without a breast in some African cultures can be influenced by a combination of religious, cultural, and gender‐related factors. Many women fear being perceived as “less of a woman” if they undergo breast removal. Several studies included in our review indicate that religious beliefs are frequently associated with delays in timely diagnosis among both Muslim and Christian women, although adherence varies by community. It is important to interpret these findings as associations rather than causal effects, as such delays may also be influenced by socioeconomic factors, cultural norms, or lack of access to healthcare infrastructure within these religious communities. Additionally, healthcare providers other than academically trained doctors are often the only available care facility, and population screening programs are not accessible in Africa. Developing educational programs that involve religious leaders to properly contextualize BC within faith communities while emphasizing the importance of medical treatment is needed. Training providers to address religious beliefs supports timely care.^65^, ^66^ Efforts include myth‐busting campaigns, health education by religious leaders, and culturally sensitive training for healthcare workers.^67^

### 5.5 Comparison of diagnostic delay individual‐level factors between high‐income countries and African countries

As the current review expands on the work of Hoveling et al.,^16^ comparison between high‐ and low‐income settings shows key differences. Both settings share common challenges, yet differences reflect the distinct healthcare, cultural, and socioeconomic contexts. Psychological barriers, fear, and stigma are key contributors to delays in both high‐income countries and African countries. In both contexts, patients often delay seeking care due to fear of a cancer diagnosis, concerns about treatment side effects, or misjudging the severity of their symptoms. Competing responsibilities are also factors hindering timely help‐seeking. Across both regions, misinformation and low health literacy remain common, contributing to delays in recognizing symptoms and seeking appropriate care. Financial barriers exist globally but are far more pronounced in African countries, where high out‐of‐pocket costs and limited healthcare infrastructure in rural areas increase delay. In these regions, delays are frequently driven by lack of awareness and knowledge about BC, leading to misinterpretation of symptoms. On the other hand, in high‐income countries, financial challenges are often linked to systemic issues, such as underinsurance or high co‐pays, while logistical barriers like long waiting times for specialist appointments also play a role. In high‐income countries, supportive family or community networks generally encourage early medical consultation, though misinformation within these networks can also perpetuate delays. In African countries, however, societal norms and family dynamics often discourage early help‐seeking. Women may delay diagnosis due to fears of stigma, divorce, or the burden of prioritizing their roles as mothers and caregivers. Still, support from husbands and leaders can promote care. Cultural and systemic barriers also show contrasts. In high‐income countries, logistical challenges like limited telehealth access and immigration‐related issues are prominent, while in African countries, reliance on healthcare providers other than academically trained doctors, distrust in modern healthcare, and spiritual beliefs about illness delay timely diagnosis. Additionally, a significant systemic barrier is the high participation rate in population screening programs in high‐income countries, while such screening programs are generally absent in African countries.

### 5.6 Strengths and limitations

A strength of this review is its narrow focus on African countries. By concentrating on this specific geographical region, the review primarily offers a deeper and comprehensive understanding of challenges and factors affecting cancer diagnosis in African communities, such as differences in access to medical resources, cultural beliefs and fears, and educational factors. However, while our review highlights individual‐level factors, we recognize that these delays are also influenced by systemic healthcare constraints, such as shortages of trained professionals and diagnostic resources. Given that health system analysis falls outside our area of expertise, we deliberately focused on individual factors. We applied Bronfenbrenner's ecological systems theory, allowing us to consider influences beyond the individual level, such as the role of immediate social networks. This approach helps health care providers, policy makers, and researchers develop tailored interventions that better address the specific barriers faced by women in these countries. In addition, this approach can help develop tailored interventions that are more likely to be effective in addressing the specific barriers faced by women in these countries. Incorporating Bronfenbrenner's ecological model to analyze the structure of influences on delay is a second strength of this research. This theoretical framework enables a comprehensive categorization of factors contributing to diagnostic delays at multiple levels. By systematically exploring how individual behaviors and experiences are shaped by wider social, cultural, and environmental contexts, our review offers a structured comparison with data from high‐income countries analyzed in a similar way. Specifically, while prior reviews have provided valuable general insights into factors contributing to diagnostic delay, our review places a stronger emphasis on individual‐level factors and their interactions with broader contextual influences. However, we intentionally conducted a scoping review rather than a meta‐analysis, as our aim was to provide a broad overview rather than a statistical synthesis. Due to study heterogeneity, quantification was not feasible. A meta‐analysis could be a next step to refine our findings further. We suggest that future research benefits from developing a standardized questionnaire to systematically analyze these factors across studies. Our synthesis provides a comprehensive overview to guide future improvements in interventions and study designs.

However, three limitations of this study should be considered when further interpreting and applying the results. Firstly, recognizing the generalizability within the African context when reflecting on factors contributing to diagnostic delay is important. From a total of 54 existing African countries, this study only investigated studies from 14 of these countries. Although African countries are highly similar in their healthcare system and cultural beliefs, making inferences about countries that were not included should be undergone carefully. Particularly in the samples from Ghana, another bias could have occurred. Three of the four studies from Ghana recruited patients from the same patient center. While these studies were executed at different times, it is still necessary to consider that these results only partly reflect the Ghanaian population. Reliance on literature may introduce bias, as variations in study quality and methodology could affect the robustness of the conclusions. Additionally, our scoping review did not assess the timeliness of diagnostic delay, which can vary across studies and healthcare settings. Definitions of diagnostic delay differ, the WHO Global Breast Cancer Initiative, for example, has set a target of a diagnostic interval of less than 60 days, but adherence to this target varies widely.^7^

## 6 CONCLUSION

Diagnostic delays in women with BC in Africa are caused by multifaceted factors across individual, family, community, and broader societal levels. Our review shows that low awareness, cultural beliefs, reliance on healthcare providers other than academically trained doctors, and religious perspectives highly contribute to delayed diagnosis. Given the lower life expectancy in many African countries, the average age at BC diagnosis is likely to be younger than in EU or other high‐income countries. However, it is important to note that estimating age‐standardized mortality rates in many African countries is challenging due to the limited availability of high‐quality data. Although estimates suggest only marginally elevated age‐standardized mortality rates for Africa, these rates do not fully capture the extreme patient mortality rates, which reflect the stark inequities in healthcare and patient survival across the continent. As life expectancy in these regions continues to rise, and considering that BC primarily affects women at older ages, the incidence of BC is expected to increase further in African countries. This makes targeted interventions and effective strategies even more important. Existing interventions need expansion and better integration. Collaborating with religious leaders could reduce stigma and promote early treatment, as religious beliefs often play a key role in healthcare decisions in African regions. Educational programs that involve religious leaders as key stakeholders, alongside culturally sensitive training for healthcare workers, are important to improve early diagnosis and treatment of BC. Furthermore, implementing population screening programs, similar to those in high‐income countries, could accelerate BC diagnostics. Future research should continue to explore diagnostic delays across different African and other low‐ and middle income contexts, focusing on gender‐specific and culturally tailored strategies to enhance early detection and improve BC and general cancer outcomes. Diagnostic delay alone does not explain poor survival. Treatment‐related barriers, such as limited access to oncology services and financial constraints, also play a role in BC outcomes. Future research should also explore how diagnostic delays interact with barriers to treatment completion to provide a more comprehensive understanding of BC survival in Africa.

## AUTHOR CONTRIBUTIONS

**Liza A. Hoveling:** Conceptualization; investigation; writing – original draft; methodology; validation; visualization; writing – review and editing; formal analysis; project administration; data curation. **Lynn P. Heuken:** Writing – original draft; visualization; formal analysis. **Thachita Harfst:** Writing – original draft; visualization; formal analysis. **Melinda S. Schuurman:** Writing – original draft; visualization; writing – review and editing; formal analysis. **Kristel M. van Asselt:** Conceptualization; investigation; writing – review and editing; methodology; validation; data curation. **Sabine Siesling:** Conceptualization; investigation; methodology; validation; writing – review and editing; data curation; supervision. **Christina Bode:** Conceptualization; investigation; methodology; validation; writing – review and editing; data curation; supervision.

## CONFLICT OF INTEREST STATEMENT

The authors declare no conflicts of interest.

## Supporting information


**Data S1.** Tables information.

## Data Availability

All results are available in Table S3. Further information is available from the corresponding author upon reasonable request.
