# Supplementary material for: Understanding delays in breast cancer diagnosis in Africa: Key insights and contributing factors
Source: Int J Cancer. 2025 Jun 20;157(9):1830–40. doi: 10.1002/ijc.70008 (PMC12407049; doi:10.1002/ijc.70008)
Supplement: Supplementary file 1 — Data S1. Tables information. [file IJC-157-1830-s001.pdf]

Supplementary Tables

Liza A. Hoveling, Lynn P. Heuken, Thachita Harfst, Melinda S. Schuurman, Kristel M. van Asselt, Sabine Siesling and Christina Bode

Table of contents

Supplementary Table 1. Keyterms Pubmed 2
Supplementary Table 2. Keyterms Scopus 3
Supplementary Table 3. Study characteristics and factors related to diagnostic delay in women with breast cancer in Africa 4
Supplementary Table 4. Characteristics of included studies on diagnostic delay in women with breast cancer in Africa 14

Supplementary Table 1. Keyterms Pubmed

#	Keyterm	Search string	Total hits
1	Diagnostic delay	(((diagnos*[Title/Abstract]) AND (delay*[Title/Abstract])) OR ((care[Title/Abstract]) AND (delay*[Title/Abstract])) OR "patient* delay*" [Title/Abstract] OR "presentation delay*" [Title/Abstract] OR "timely diagnos*" [Title/Abstract] OR "primary care delay*" [Title/Abstract] OR "late diagnos*" [Title/Abstract])	158,774
2	Cancer	(cancer [Title/Abstract] OR tumor [Title/Abstract] OR neoplasm [Title/Abstract] OR malignan* [Title/Abstract] OR carcino* [Title/Abstract] OR oncolog* [Title/Abstract] OR sarcoma [Title/Abstract] OR leukemia [Title/Abstract] OR lymphoma [Title/Abstract] OR melanoma [Title/Abstract] OR blastoma [Title/Abstract])	4,104,394
3	Factors	(determinant* [Title/Abstract] OR influence* [Title/Abstract] OR barrier* [Title/Abstract] OR factor* [Title/Abstract] OR reason* [Title/Abstract])	6,419,852
4	Women	(women [Title/Abstract] OR woman [Title/Abstract] OR female [Title/Abstract] OR gender [Title/Abstract] OR sex [Title/Abstract] OR breast [Title/Abstract] OR cervi* [Title/Abstract] OR uter* [Title/Abstract] OR endometr* [Title/Abstract] OR ovar* [Title/Abstract] OR vulv* [Title/Abstract])	3,827,663
5		#1 AND #2 AND #3 AND #4	3,690

Supplementary Table 2. Keyterms Scopus

#	Keyterm	Search string	Total hits
1	Diagnostic delay	TITLE-ABS-KEY((diagnosis AND delay) OR (care AND delay) OR "patient delay" OR "presentation delay" OR "timely diagnosis" OR "primary care delay" OR "late diagnosis")	128,799
2	Cancer	TITLE-ABS-KEY(cancer OR tumor OR neoplasm OR malignant OR carcinoma OR oncology OR sarcoma OR leukemia OR lymphoma OR melanoma OR blastoma)	6,549,118
3	Factors	TITLE-ABS-KEY(determinant OR influence OR barrier OR factor OR reason)	16,703,664
4	Women	TITLE-ABS-KEY(women OR woman OR female OR gender OR sex OR breast OR cervix OR uterus OR endometrium OR ovary OR vulva)	13,510,247
5		#1 AND #2 AND #3 AND #4	7,807

Supplementary Table 3. Study characteristics and factors related to diagnostic delay in women with breast cancer in Africa

Title	Author/year	Country	Self-screen detected (yes) or symptomatic (no)	Qualitative or quantitative study	Aims of the study	Study population	Study centre	Year of population inclusion	Study design	Type of delay	Individual factors	Most important individual factors
Breast cancer awareness and screening practice amongst health personnel and general population of the littoral region of Cameroon	Halmata et al. - 2021	Cameroon	No	Quantitative	This study aimed at evaluating the awareness of BC, its warning signs, and screening methods among healthcare professionals and the general population of Douala.	737 participants (121 health personnel and 616 women)	Six health facilities in the city of Douala, Littoral Region, Cameroon	June to October 2017	Cross-sectional and descriptive study	Diagnostic delay	Awareness on BCs prevention, screening methods, attitudes regarding screening, methods use in case of nodule in patient, frequency of breast self-examination practice	Lack of awareness, insufficient knowledge and misperceptions on its risk factors and causes of professionals and low practice of BC screening amongst women in Douala and highlight the need to raise awareness and provide the right information to the public for early detection of BC
Awareness of breast cancer Screening among the Medical and General Population of the North Region of Cameroon	Simo et al. - 2021	Cameroon	No	Quantitative	This study aimed to evaluate BC awareness in the North Region of Cameroon.	475 women randomly selected among the residents of the rural areas around the city of Garoua, North Region, Cameroon	Six health facilities of the North Region of Cameroon	August to December 2019	Cross-sectional	Diagnostic delay	Awareness of BC risk factors. Information source: media, friends, health professionals, school. Risk factors: none, hereditary, prolonged use of pills, alcohol and tobacco, age, obesity, prolonged exposure to the sun, low breastfeeding, low deliveries, traditional breast massage, extended wearing of tight bra. Symptoms: none, lump of the breast, pain in the breast, wound on the breast, changes in breast shape and appearance, nipple discharge	Out of the 475 women (including 37 medical personnel) interviewed, 45.5% attended at least secondary school; 91.3% were aware of the disease with the main sources of information from those around them (64.8%), media (46.5%), and health professionals in health facilities (42.7%). 23.3% had misconceptions and myth-based ideas on the origin of the disease. Ignorance was the main reason preventing the performance of breast self-examination, and the high cost prevents individuals from going for mammography
Factors associated with late-stage diagnosis of breast cancer among Egyptian women	Ismail et al. - 2021	Egypt	Both	Quantitative	This study aimed to identify the factors linked to the late-stage diagnosis of BC in Egypt.	400 BC patients	The main tertiary cancer hospitals in Egypt.	-	Cross-sectional	Diagnostic delay	Age, marital status, place of residence, level of education, occupation, income	Marital status

Perspectives of patients, family members, and health care providers on late diagnosis of breast cancer in Ethiopia: A qualitative study	Gebremariam et al. 2019a	Ethiopia	No	Qualitative	This study aimed to explore the main reasons for diagnosis of advanced stage BC from the perspective of patients, family members, and health care providers.	13 BC patients, 5 family members, and 5 health care providers.	Tikur Anbessa Specialized Hospital (TASH) Oncology Center.	from March–July 2017	A phenomenological study	Diagnostic delay	Lack of awareness about BC in the community, disregarding or misattribution of BC symptoms, misperceptions about BC treatment and its outcomes, non-medical management of BC symptoms, fear of cancer diagnosis, competing priorities, financial insecurity, health system-related barriers. Facilitators of early diagnosis of BC: persuasion by family. members and friends, prior knowledge of someone with BC, the literacy level of the women	Lack of awareness, Disregarding, or misattribution of BC symptoms, Misperceptions about BC treatment and its outcomes, Non-medical management of BC symptoms, Health system-related barriers
Why women with breast cancer presented late to health care facility in North-West Ethiopia? A qualitative study	Tesfaw et al. - 2020a	Ethiopia	No	Qualitative	This study aimed to explore the reasons for patient delay in seeking early medical care for BC in North-West Ethiopia	Newly diagnosed BC women in two hospitals of North-West Ethiopia	Two specialized hospitals in North West Ethiopia	From November to December 2019	In-depth interviews	Patient delay	Lack of knowledge and awareness about BC, cultural and religious beliefs, economic hardships, lack of health care and transportation access, fear of surgical procedures and lack of trusts on medical care were the major reasons for late presentation of BC identified from the patient's narratives	Lack of knowledge and awareness
Time intervals experienced between first symptom recognition and pathologic diagnosis of breast cancer in Addis Ababa, Ethiopia: a cross-sectional study	Gebremariam et al. 2019b	Ethiopia	No	Quantitative	This study aimed to estimate the magnitude of patient and diagnostic delays and associated factors among women with BC in Addis Ababa	441 BC patients	Seven major healthcare facilities in Addis Ababa	20 March 2017 and ended on 31 July 2018	Cross-sectional	Patient delay and diagnostic delay	Age at diagnosis, highest level of education, marital status, occupation, family size, family monthly income, source of medical expenses, consultation after recognition of symptom progression	Consultation after recognition of symptom progression.
Association of Delay in Breast Cancer Diagnosis With Survival in Addis Ababa, Ethiopia: A Prospective Cohort Study.	Gebremariam et al. 2023	Ethiopia	No	Quantitative	This study aimed to examine the association between delayed BC diagnosis and overall survival among women in Addis Ababa, Ethiopia.	439 BC patients	Seven major health facilities in Addis Ababa, Ethiopia	January 1, 2017, to June 30, 2018,	Baseline questionnaire and 3 calls per day	Diagnostic delay	Education, marriage, traditional medicine, and stage of disease	Less educated, unmarried, use traditional medicine, and present with advanced-stage disease.

Patient Delay and Contributing Factors Among Breast Cancer Patients at Two Cancer Referral Centres in Ethiopia: A Cross-Sectional Study	Tesfaw et al. - 2020b	Ethiopia	No	Quantitative	This study aims to investigate patient delay in BC presentation and identify contributing factors to diagnosis in Ethiopia, where high mortality is linked to prolonged delays and advanced-stage presentations.	371 BC patients	Two oncology units of the University of Gondar and Felege Hiwot specialized hospitals	September 2019 to April 30, 2020	Cross-sectional	Patient delay and diagnostic delay	Travel distance ≥5 km, rural residence, no history of any breast problem before, having no lump/swelling in the arm pit, a painless lump in the breast, and being illiterate	Travel distance and lack of symptoms
Late-Stage Diagnosis and Associated Factors Among Breast Cancer Patients in South and Southwest Ethiopia: A Multicenter Study	Tesfaw et al. - 2021	Ethiopia	No	Quantitative	This study aimed to determine the occurrence of late-stage disease and its associated factors in selected public hospitals in South and Southwest Ethiopia.	426 BC patients	Hawassa Comprehensive Referral Hospital,Attat Our Lady of Lourdes Hospital, Dubo St Mary PrimaryHospital, Wolliso St Lukas Primary Hospital, Durame GeneralHospital, and Butajira General Hospital	January 1, 2013 to December 31, 2017	Cross-sectional	Diagnostic delay	Patient delay, health system delays, female sex, rural residence, chief complaint of breast lump, history of comorbidities	Patient delay, health system delays, female sex, rural residence, chief complaint of breast lump, history of comorbidities
Seeking healthcare at their 'right' time; the iterative decision process for women with breast cancer	Agbeko et al. - 2020	Ghana	No	Qualitative	This study aimed to assess symptom appraisal and healthcare-seeking behaviour.	15 BC patients	Komfo Anokye Teaching Hospital, Ghana	May 2015 to March 2016	Interviews	Presentation delay	Economic, social and family functioning, education on symptom appraisal	Economic, social and family functioning, education on symptom appraisal
Knowledge and Health Seeking Behaviour of Breast Cancer Patients in Ghana	Agbokey et al. - 2019	Ghana	No	Qualitative	This study aimed to explore the knowledge and healthcare-seeking behaviour of patients.	35 BC patients	Komfo Anokye Teaching Hospital, Ghana	June 14th - July 24th 2014	Cross-sectional	Patient delay	Infrequent self-screening, low knowledge for risk factors even after diagnosis, reported late due to misinterpretation of signs and symptoms, cultural influences and fear of losing their breast to surgery, physician delay, health providers' laxity, and disinterest in BC, husbands/men decided on	Low knowledge

											treatment details, , went delayed to hospitals	
Recognizing and appraising symptoms of breast cancer as a reason for delayed presentation in Ghanaian women: A qualitative study	Bonsu et al. - 2019	Ghana	No	Qualitative	This study aimed to identify factors contributing to delayed presentation.	11 BC patients	Komfo Anokye Teaching Hospital, Ghana	January, 2018 through to June, 2018	In- depth interviews	Delayed presentation	Symptom experience, knowledge, role of social life/network, coping with symptoms, intent to seek health care	Symptom experience, knowledge, role of social life/network, coping with symptoms, intent to seek health care
Fear of Mastectomy Associated with Delayed Breast Cancer Presentation Among Ghanaian Women	Martei et al. - 2018	Ghana	No	Qualitative	This study aimed to characterize sociocultural factors associated with delayed presentation.	31 BC patients	Korle Bu Teaching Hospital in Accra, Ghana	June 2008 and August 2008	In-depth interviews	Delayed presentation	Fear of mastectomy due to stigma, church gave social stigma to BC patients, awareness but lack of knowledge, fail to associate "painless" breast with breast malignancy, financial burden of treatment	Fear of mastectomy
Identifying Barriers and Facilitators to Breast Cancer Early Detection and Subsequent Treatment Engagement in Kenya: A Qualitative Approach	Gakunga et al. - 2019	Kenya	No	Qualitative	This study aimed to expand the existing understanding of barriers and facilitators in accessing BC care.	Four focus groups with each 6-11 women, classified groups based on diagnosis and socioeconomic status	Kenya Cancer Association and Kenya Hospices and Palliative Care Association, and their affiliates	February and June 2018	Focus groups and hermeneutic (interpretive) phenomenolog y	Barriers and facilitators in BC care	Costs, inadequate knowledge, distance to health facilities, communication with health providers, medicines stockouts, long waiting periods, limited to no counselling at diagnosis, patient vulnerability, limited access to rehab items (facilitators: social support, periodical access to awareness and early detection services, friendly caregiver; no effect at all from socioeconomic status)	Costs, inadequate knowledge, distance to health facilities, communication with health providers, medicines stockouts, long waiting periods, limited to no counselling at diagnosis, patient vulnerability, limited access to rehab items (facilitators: social support, periodical access to awareness and early detection services, friendly caregiver; no effect at all from socioeconomic status)
Delayed breast cancer presentation, diagnosis, and treatment in Kenya	Daniel et al. - 2023	Kenya	No	Qualitative + Quantitative	This study aimed to evaluate the factors contributing to diagnostic delay and treatment.	378 patients with a diagnosis of BC	Kenyatta National Hospital	-	Mixed method approach: cross-sectional questionnaires and in-depth interviews	Diagnostic and treatment delay	Never/unmarried, less educated, less affluent, users of hormonal contraception, and had ≥ 3 children were more likely to experience diagnosis and treatment delay, financial constraints, lack of patient BC awareness, and healthcare practitioner misdiagnosis and/or strikes	Educating communities and providers about BC

Why do Women with Breast Cancer Get Diagnosed and Treated Late in Sub-Saharan Africa? Perspectives from Women and Patients in Bamako, Mali	Grosse Frie et al. - 2018	Mali	No	Qualitative	This study aimed to analyse barriers throughout a BC patient's pathway from symptom recognition to treatment in Mali.	One discussion with 8 BC survivors at a local non-government organization, and two discussions – one with 12 and one with 5 (healthy) women – in 2 of the 6 communities in Bamako.	Medical Faculty in Bamako, Mali	Between January and February 2016	Focus group interviews	Diagnostic delay	Barriers during the appraisal interval were a low level of BC knowledge among women, their families, and medical professionals, and during the help-seeking interval, mistrust in the community health care centres and economic hardship. Barriers during the diagnosis interval were low quality of health care services and lack of social support.	Barriers during the appraisal interval were a low level of BC knowledge among women, their families, and medical professionals, and during the help-seeking interval, mistrust in the community health care centres and economic hardship. Barriers during the diagnosis interval were low quality of health care services and lack of social support.
Sociocultural Barriers Related to Late-Stage Presentation of Breast Cancer in Morocco	Soliman et al. - 2018	Morocco	No	Qualitative	This study aimed to investigate sociocultural barriers contributing to delayed presentation and diagnosis of BC among women in Marrakesh, Morocco.	25 BC patients	CHU Mohammed VI Hospital in Marrakesh	June to July 2014	Interviews	Diagnostic delay	Treatment-associated costs, burden of transportation and distance, choice of medical arena, identity and femininity, community influence, spirituality and conception of death	Structural barriers included high treatment-associated costs for patients and their families, burden of transportation to central treatment centres, and limited access to appropriate health care resources. Sociocultural barriers included perceived attack on one's identity associated with BC diagnosis and treatment, influence of the local community, and ideas of faith, spirituality, and conception of death
The patient, diagnosis and treatment delay of the care pathway of patients with breast cancer in Morocco	Mimouni et al. - 2022	Morocco	No	Quantitative	This study aimed to document the time intervals of BC, particularly delays in patient presentation, diagnosis, and treatment, and to evaluate how clinical, socio-demographic, and treatment factors influence these delays throughout the clinical course.	410 BC patients	FES hospital of oncology in Morocco	Years 2013–2017	Cross-sectional	Patient, diagnostic and treatment delay	Age, profession, nature of the consultation, family history of cancer, medical coverage, diagnosis year, stage histologic tumour, biopsy, pre-centre investigation, stage UICC	Patient delay: diagnosis year. Diagnosis delay: age and diagnosis year.

Factors Related to Delayed Diagnosis and Treatment of Breast Cancer Among Moroccan Women in Casablanca	Zahfir et al. - 2022	Morocco	Both	Quantitative	This study aimed at investigating the factors associated with delayed diagnosis and treatment of BC in Casablanca	300 BC patients	University hospital center Ibn Rochd of Casablanca and the private center Ryad of Casablanca	January to June 2018	Cross-sectional	Diagnostic delay and treatment delay	Age, professional status, residence, educational level, marital status, monthly income, type of coverage medical, distance from medical care, medical care, family history of cancer, knowledge about breast self-examination, breastfeeding, mode of discovery and breast pain as initial symptom.	Marriage and consulting once or twice before diagnosis in public care
Effect of sociodemographic variables on patient and diagnostic delay of breast cancer at the foremost health care institution in Nigeria	Olarewaju et al. - 2019	Nigeria	No	Quantitative	This study aimed to investigate the types and extent of delays in BC diagnosis at Nigeria's leading hospital, while also identifying the key factors contributing to these delays.	275 BC patients	University College Hospital, Ibadan, Nigeria	August to October 2018	Cross-sectional	Patient and diagnostic delay	Patient delay: age, ethnicity, religion, marital status, education, employment, income. Diagnostic delay: age, ethnicity, marital status, religion, marital status, education, employment, income.	Patient delay: ethnicity, marital status. Diagnostic delay: marital status
Delayed help-seeking for symptomatic breast cancer: reasons for delay among participants receiving treatment at a public healthcare facility in South Africa	Swinny et al. - 2021	South Africa	No	Qualitative	This study aimed to examine the perceived personal and structural barriers to seeking medical help among women with BC in this low-resource setting.	25 BC patients	Division of Radiation Oncology at largest hospital in South Africa	-	Interviews	Delayed help seeking behaviour (patient/presentation delay)	Participants' limited knowledge of BC, negative views of the healthcare system, and challenging life experiences, such as marital discord, perceived lack of support, and caring for sick loved ones, contributed to their help-seeking delays	Knowledge on BC and their own stages
Barriers to early presentation of breast cancer among women in Soweto, South Africa	Joffe et al. - 2018	South Africa	No	Quantitative	This study aimed to identify patient- and provider-related factors associated with the clinical stage at diagnosis among women.	499 BC patients	Chris Hani Baragwanath Academic Hospital in Soweto, Johannesburg	2015–2016	Face-to-face interviewer-administered questionnaires	Early and late stage diagnosis	Age, educational level, household socioeconomic, HIV status, treated hypertension, BMI, intrinsic receptor subtype, knowledge and awareness, time to 1st visit to health system, number of HS visits, referral pathway to hospital.	Limited education, knowledge and awareness, health system inefficiency

Unraveling the South African Breast Cancer Story: the Relationship of Patients, Delay to Diagnosis, and Tumor Biology With Stage at Presentation in an Urban Setting	Rayne et al. - 2019a	South Africa	No	Quantitative	This study aimed to investigate the stage of disease at presentation in an urban South African population and assess the relative contributions of patient characteristics, treatment delay, and tumour biology.	231 BC patients	An urban South African open-access breast care clinic	January 2016-February 2017	Questionnaire and file review	Diagnostic delay	Age, self-reported race, family history, comorbidities, total time to presentation, molecular subtype, histological subtype, histological grade, immunohistochemistry	Less than 45 years, black African, luminal B, triple negative
The effect of beliefs about breast cancer on stage and delay to presentation: results from a prospective study in urban South Africa	Rayne et al. - 2019b	South Africa	No	Quantitative	This study aimed to examine the relationship between attitudes and beliefs about BC and the stage at diagnosis, as well as delays in diagnosis, in South Africa.	233 BC patients	Clinics in South Africa	-	Cross-sectional	Diagnostic delay	Questions asked: This cancer is a punishment, this is a curse from God, someone has cursed me with this cancer, people with cancer will die when they go to hospital, I would rather die than lose my breast, it is better to die with a breast than to be less than a whole women, the doctors will leave me without my breast, I can't afford to get cancer, only old women get BC, BC can be caught from others, there is no BC in my family so I shouldn't have got cancer, people have been cured of cancer by traditional non-medical healing alone, people have been cured of cancer using alternative therapies alone, there are many ways BC can be treated, not just through medicine and surgery alone, people have been cured of cancer through prayer and faith alone, my faith in God alone will heal me, I can look after myself, my family will support me, I [do not] believe that cancer will kill me, I believe I can beat cancer, until I get cured, beating my cancer comes first in my life,<br>I believe that if someone gets cancer... 1)...their time to die is near, 2)...that's the way they were meant to die, 3)...a lot of different treatments won't work, 4)...it doesn't matter they found out about it, they will still die, 5)...cancer kills most people who get it,	Most women believed cancer could be beaten (90.0%), and their families would support them (92.8%). They disagreed that cancer was a curse (93.8%), punishment (90.5%) or that alternative therapies or traditional healing would cure their cancer (75.3% and 85.5% respectively). On univariate analysis, age under 45 years and transport difficulties predicted advanced stage at presentation. No socio-economic factors or beliefs increased the risk of delay to presentation

											6)...it is part of god's plan, 7)...it was meant to be	
Delay to diagnosis and breast cancer stage in an urban South African breast clinic	Rayne et al. - 2019c	South Africa	No	Quantitative	This study aimed to identify the points at which patient-related socioeconomic delays occur and how they relate to the stage at presentation.	252 BC patients	Few specialist breast-care clinics in South Africa	January 2016 to February 2017	Cross-sectional	Patient delay	Age, marital status, education, family history, dependants, cell phone, internet use, employment status, transport to breast clinic, travel to breast clinic, fears about missing appointments, total delay to presentation at breast clinic, delay to acknowledgement, delay to any health facility, delay to specialist breast clinic	Transport to breast clinic, cell phone, total delay to presentation at breast clinic
Delayed diagnostic evaluation of symptomatic breast cancer in sub-Saharan Africa: A qualitative study of Tanzanian women	Sakafu et al. - 2022	Tanzania	No	Qualitative	This study aimed to identify factors contributing to delayed diagnostic evaluation among women with BC in Tanzania.	12 BC patients	Muhimbili National Hospital in Dares Salaam, Tanzania	June to September 2019	In-depth interviews	Diagnostic delay	Lack of basic knowledge and awareness of BC and misconceptions, barriers with their local primary healthcare providers, including symptom mismanagement and delayed referrals for diagnostic evaluation. Other barriers included financial hardships, fear and stigma of cancer, and use of traditional medicine. The advice and influence of family members and friends played key roles in healthcare-seeking behaviours, serving as both facilitators and barriers	Lack of basic knowledge and awareness of BC, stigma, financial barriers, and local healthcare system barriers
Factors associated with late diagnosis of breast cancer in women in Togo, Sub-Saharan Africa	Darre et al. - 2023	Togo	No	Quantitative	This study aimed to identify factors associated with the late diagnosis of BC in Togolese women.	62 BC patients	Laboratory of Pathological Anatomy and Cytology of the CHU Sylvanus Olympia	2021	Cross-sectional	Diagnostic delay	Distance from health care facilities, fear of diagnosis, diagnostic error, lack of financial means, type of first consultation, breast self-examination	Fear of diagnosis, type of first consultation, breast self-examination

Factors Associated with Diagnostic Delays Among Tunisian Breast Cancer Patients	Balhi et al. - 2023	Tunisia	No	Quantitative	This study aimed to analyse factors associated with delayed presentation among women.	146 BC patients	Salah Azaeiz institute of Tunis	January 1st to June, 30th, 2021	Retrospective cohort study	Patient delay, healthcare system delay	Age, marital status, educational level, professional status, residence areas, distance from hospital, first symptom of discovery, reasons not to consult, traditional healer consulted	Lack of knowledge about symptoms
Factors associated with diagnostic and pre-treatment intervals among breast cancer patients attending care at the Uganda Cancer Institute: A cross-sectional study	Achan et al. - 2023	Uganda	No	Quantitative	This study aimed to assess the diagnostic and pre-treatment intervals and identify factors associated with these intervals among BC patients receiving care at the Uganda Cancer Institute (UCI).	401 BC patients	Uganda Cancer Institute (UCI).	Between June and October 2019	Cross-sectional	Diagnostic delay	Marital status, age group, education level, employment status, region of origin in Uganda, family history of BC, having prior information on BC, risk factor knowledge level, distance from home to nearest health facility, distance from home to Uganda Cancer Institute, number of visits to health facility before referral to Uganda Cancer Institute, level of referring health facility.	Tertiary education level, distance from home to UCI

Factors leading to the late diagnosis and poor outcomes of breast cancer in Matabeleland South and the Bulawayo Metropolitan Provinces in Zimbabwe	Magara et al. - 2023	Zimbabwe	No	Qualitative + Quantitative	This study aimed to identify key factors delaying the diagnosis and treatment of women with suspected BC in Matabeleland South and Bulawayo Metropolitan Provinces. The primary objective is to highlight the most significant and potentially correctable causes of delay and explore opportunities to address them.	Group 1. A total of 1250 questionnaires were distributed to women (patients) and their relatives in the MS Province districts and Bulawayo Metropolitan Province. Returns numbered 1107 respondents (return rate 88.56%). Group 2. A total of 414 questionnaires were administered; of those 298 were completed and returned (return rate of 71.98%)	Mpilo Central Hospital in Bulawayo is the only facility providing Radio- therapy, Oncology and Histopathology services for the whole region	-	Mixed method approach: cross-sectional questionnaires and focus group discussions	Diagnostic delay	Key messages identified: The lack of BC awareness in the community reflects the historical neglect of education, public health initiatives and national campaigns, Ignorance, fear and cultural factors combine with poor services/equipment to delay diagnosis, Traditional is more popular than conventional medicine particularly in rural communities, until BC becomes advanced, Travelling long distances for treatment is a major problem for many patients, The high costs of conventional BC investigation and treatment are unaffordable for most women living in poor communities, Families in rural communities play a key role in decisions about the location and type of treatment, Lack of standardised information and data collection combine with poor interprofessional communication to undermine trust in conventional treatment	Younger women (<30years) had the least knowledge of BC. Analysis confirmed a strong association between age and awareness of BC incidence with respondents aged 30–39 years being both the largest group represented and the least knowledgeable, independent of speciality. Nearly all respondents (90%) supported decentralisation of appropriate breast surgical services to provincial and district hospitals backed up by specialist training. Thematic analysis of focus group discussions identified the following as important contributors to late BC diagnosis and poor outcomes: (i) presentation is delayed by poorly educated women and their families who fear BC and high treatment costs (ii) referral is delayed by health professionals with no access to training, skills or diagnostic equipment (iii), treatment is delayed by disorganised, over-centralized patient pathway, and a lack of specialist care and inter-disciplinary communication

Supplementary Table 4. Characteristics of included studies on diagnostic delay in women with breast cancer in Africa

Title	Author/year	Country	Aims of the study	Study population	Study design	Type of delay
Breast cancer awareness and screening practice amongst health personnel and general population of the littoral region of Cameroon	Halmata et al. - 2021	Cameroon	This study aimed at evaluating the awareness of BC, its warning signs, and screening methods among healthcare professionals and the general population of Douala.	737 participants (121 health personnel and 616 women)	Cross-sectional and descriptive study	Diagnostic delay
Awareness of breast cancer Screening among the Medical and General Population of the North Region of Cameroon	Simo et al. - 2021	Cameroon	This study aimed to evaluate BC awareness in the North Region of Cameroon.	475 women randomly selected among the residents of the rural areas around the city of Garoua, North Region, Cameroon	Cross-sectional	Diagnostic delay
Factors associated with late-stage diagnosis of breast cancer among Egyptian women	Ismail et al. - 2021	Egypt	This study aimed to identify the factors linked to the late-stage diagnosis of BC in Egypt.	400 BC patients	Cross-sectional	Diagnostic delay
Perspectives of patients, family members, and health care providers on late diagnosis of breast cancer in Ethiopia: A qualitative study	Gebremariam et al. 2019a	Ethiopia	This study aimed to explore the main reasons for diagnosis of advanced stage BC from the perspective of patients, family members, and health care providers.	13 BC patients, 5 family members, and 5 health care providers.	A phenomenological study	Diagnostic delay
Why women with breast cancer presented late to health care facility in North-West Ethiopia? A qualitative study	Tesfaw et al. - 2020a	Ethiopia	This study aimed to explore the reasons for patient delay in seeking early medical care for BC in North-West Ethiopia	Newly diagnosed BC women in two hospitals of North-West Ethiopia	In-depth interviews	Patient delay
Time intervals experienced between first symptom recognition and pathologic diagnosis of breast cancer in Addis Ababa, Ethiopia: a cross-sectional study	Gebremariam et al. 2019b	Ethiopia	This study aimed to estimate the magnitude of patient and diagnostic delays and associated factors among women with BC in Addis Ababa	441 BC patients	Cross-sectional	Patient delay and diagnostic delay
Association of Delay in Breast Cancer Diagnosis With Survival in Addis Ababa, Ethiopia: A Prospective Cohort Study.	Gebremariam et al. 2023	Ethiopia	This study aimed to examine the association between delayed BC diagnosis and overall survival among women in Addis Ababa, Ethiopia.	439 BC patients	Baseline questionnaire and 3 calls per day	Diagnostic delay
Patient Delay and Contributing Factors Among Breast Cancer Patients at Two Cancer Referral Centres in Ethiopia: A Cross-Sectional Study	Tesfaw et al. - 2020b	Ethiopia	This study aims to investigate patient delay in BC presentation and identify contributing factors to diagnosis in Ethiopia, where high mortality is linked to prolonged delays and advanced-stage presentations.	371 BC patients	Cross-sectional	Patient delay and diagnostic delay
Late-Stage Diagnosis and Associated Factors Among Breast Cancer Patients in South and Southwest Ethiopia: A Multicenter Study	Tesfaw et al. - 2021	Ethiopia	This study aimed to determine the occurrence of late-stage disease and its associated factors in selected public hospitals in South and Southwest Ethiopia.	426 BC patients	Cross-sectional	Diagnostic delay
Seeking healthcare at their 'right' time; the iterative decision process for women with breast cancer	Agbeko et al. - 2020	Ghana	This study aimed to assess symptom appraisal and healthcare-seeking behavior.	15 BC patients	Interviews	Presentation delay
Knowledge and Health Seeking Behaviour of Breast Cancer Patients in Ghana	Agbokey et al. - 2019	Ghana	This study aimed to explore the knowledge and healthcare-seeking behavior of patients.	35 BC patients	Cross-sectional	Patient delay
Recognizing and appraising symptoms of breast cancer as a reason for delayed	Bonsu et al. - 2019	Ghana	This study aimed to identify factors contributing to delayed presentation.	11 BC patients	In- depth interviews	Delayed presentation

presentation in Ghanaian women: A qualitative study						
Fear of Mastectomy Associated with Delayed Breast Cancer Presentation Among Ghanaian Women	Martei et al. - 2018	Ghana	This study aimed to characterize sociocultural factors associated with delayed presentation.	31 BC patients	In-depth interviews	Delayed presentation
Identifying Barriers and Facilitators to Breast Cancer Early Detection and Subsequent Treatment Engagement in Kenya: A Qualitative Approach	Gakunga et al. - 2019	Kenya	This study aimed to expand the existing understanding of barriers and facilitators in accessing BC care.	Four focus groups with each 6-11 women, classified groups based on diagnosis and socioeconomic status	Focus groups and hermeneutic (interpretive) phenomenology	Barriers and facilitators in BC care
Delayed breast cancer presentation, diagnosis, and treatment in Kenya	Daniel et al. - 2023	Kenya	This study aimed to evaluate the factors contributing to diagnostic delay and treatment.	378 patients with a diagnosis of BC	Mixed method approach: cross-sectional questionnaires and in-depth interviews	Diagnostic and treatment delay
Why do Women with Breast Cancer Get Diagnosed and Treated Late in Sub-Saharan Africa? Perspectives from Women and Patients in Bamako, Mali	Grosse Frie et al. - 2018	Mali	This study aimed to analyze barriers throughout a BC patient's pathway from symptom recognition to treatment in Mali.	One discussion with 8 BC survivors at a local non-government organization, and two discussions – one with 12 and one with 5 (healthy) women – in 2 of the 6 communities in Bamako.	Focus group interviews	Diagnostic delay
Sociocultural Barriers Related to Late-Stage Presentation of Breast Cancer in Morocco	Soliman et al. - 2018	Morocco	This study aimed to investigate sociocultural barriers contributing to delayed presentation and diagnosis of BC among women in Marrakesh, Morocco.	25 BC patients	Interviews	Diagnostic delay
The patient, diagnosis and treatment delay of the care pathway of patients with breast cancer in Morocco	Mimouni et al. - 2022	Morocco	This study aimed to document the time intervals of BC, particularly delays in patient presentation, diagnosis, and treatment, and to evaluate how clinical, socio-demographic, and treatment factors influence these delays throughout the clinical course.	410 BC patients	Cross-sectional	Patient, diagnostic and treatment delay
Factors Related to Delayed Diagnosis and Treatment of Breast Cancer Among Moroccan Women in Casablanca	Zahfir et al. - 2022	Morocco	This study aimed at investigating the factors associated with delayed diagnosis and treatment of BC in Casablanca	300 BC patients	Cross-sectional	Diagnostic delay and treatment delay
Effect of sociodemographic variables on patient and diagnostic delay of breast cancer at the foremost health care institution in Nigeria	Olarewaju et al. - 2019	Nigeria	This study aimed to investigate the types and extent of delays in BC diagnosis at Nigeria's leading hospital, while also identifying the key factors contributing to these delays.	275 BC patients	Cross-sectional	Patient and diagnostic delay
Delayed help-seeking for symptomatic breast cancer: reasons for delay among participants receiving treatment at a public healthcare facility in South Africa	Swinny et al. - 2021	South Africa	This study aimed to examine the perceived personal and structural barriers to seeking medical help among women with BC in this low-resource setting.	25 BC patients	Interviews	Delayed help seeking behaviour (patient/presentation delay)
Barriers to early presentation of breast cancer among women in Soweto, South Africa	Joffe et al. - 2018	South Africa	This study aimed to identify patient- and provider-related factors associated with the clinical stage at diagnosis among women.	499 BC patients	Face-to-face interviewer-administered questionnaires	Early and late stage diagnosis
Unraveling the South African Breast Cancer Story: the Relationship of Patients, Delay to Diagnosis, and Tumor Biology With Stage at	Rayne et al. - 2019a	South Africa	This study aimed to investigate the stage of disease at presentation in an urban South African population and assess the relative contributions of patient characteristics,	231 BC patients	Questionnaire and file review	Diagnostic delay

Presentation in an Urban Setting			treatment delay, and tumor biology.			
The effect of beliefs about breast cancer on stage and delay to presentation: results from a prospective study in urban South Africa	Rayne et al. - 2019b	South Africa	This study aimed to examine the relationship between attitudes and beliefs about BC and the stage at diagnosis, as well as delays in diagnosis, in South Africa.	233 BC patients	Cross-sectional	Diagnostic delay
Delay to diagnosis and breast cancer stage in an urban South African breast clinic	Rayne et al. - 2019c	South Africa	This study aimed to identify the points at which patient-related socioeconomic delays occur and how they relate to the stage at presentation.	252 BC patients	Cross-sectional	Patient delay
Delayed diagnostic evaluation of symptomatic breast cancer in sub-Saharan Africa: A qualitative study of Tanzanian women	Sakafu et al. - 2022	Tanzania	This study aimed to identify factors contributing to delayed diagnostic evaluation among women with BC in Tanzania.	12 BC patients	In-depth interviews	Diagnostic delay
Factors associated with late diagnosis of breast cancer in women in Togo, Sub-Saharan Africa	Darre et al. - 2023	Togo	This study aimed to identify factors associated with the late diagnosis of BC in Togolese women.	62 BC patients	Cross-sectional	Diagnostic delay
Factors Associated with Diagnostic Delays Among Tunisian Breast Cancer Patients	Balhi et al. - 2023	Tunisia	This study aimed to analyze factors associated with delayed presentation among women.	146 BC patients	Retrospective cohort study	Patient delay, healthcare system delay
Factors associated with diagnostic and pre-treatment intervals among breast cancer patients attending care at the Uganda Cancer Institute: A cross-sectional study	Achan et al. - 2023	Uganda	This study aimed to assess the diagnostic and pre-treatment intervals and identify factors associated with these intervals among BC patients receiving care at the Uganda Cancer Institute (UCI).	401 BC patients	Cross-sectional	Diagnostic delay
Factors leading to the late diagnosis and poor outcomes of breast cancer in Matabeleland South and the Bulawayo Metropolitan Provinces in Zimbabwe	Magara et al. - 2023	Zimbabwe	This study aimed to identify key factors delaying the diagnosis and treatment of women with suspected BC in Matabeleland South and Bulawayo Metropolitan Provinces. The primary objective is to highlight the most significant and potentially correctable causes of delay and explore opportunities to address them.	Group 1. A total of 1250 questionnaires were distributed to women (patients) and their relatives in the MS Province districts and Bulawayo Metropolitan Province. Returns numbered 1107 respondents (return rate 88.56%). Group 2. A total of 414 questionnaires were administered; of those 298 were completed and returned (return rate of 71.98%)	Mixed method approach: cross-sectional questionnaires and focus group discussions	Diagnostic delay
